# Kava constituents exert selective anticancer effects in oral squamous cell carcinoma cells in vitro

**DOI:** 10.1038/s41598-020-73058-4

**Published:** 2020-09-28

**Authors:** Antonio Celentano, Callisthenis Yiannis, Rita Paolini, Pangzhen Zhang, Camile S. Farah, Nicola Cirillo, Tami Yap, Michael McCullough

**Affiliations:** 1 Melbourne Dental School, The University of Melbourne, 720 Swanston Street, Carlton, VIC 3053 Australia; 2 School of Agriculture and Food, Faculty of Veterinary and Agricultural Sciences, The University of Melbourne, 142 Royal Parade, Parkville, VIC 3052 Australia; 3 Australian Centre for Oral Oncology Research and Education, Perth, WA 6009 Australia; 4 Oral, Maxillofacial and Dental Surgery, Fiona Stanley Hospital, Murdoch, WA 6150 Australia

**Keywords:** Cancer therapy, Oral cancer, Cancer therapy, Head and neck cancer, Oral cancer

## Abstract

Kava is a beverage made from the ground roots of the plant *Piper Methysticum*. Active compounds of Kava have previously been demonstrated to exert an antiproliferative effect through cell cycle arrest and promotion of apoptosis. Our aim was to investigate the in vitro effects of the main constituents derived from Kava on oral squamous cell carcinoma (OSCC) activity. Gas chromatography mass spectrometry (GCMS) was used to characterise the main constituents of two Kava preparations. Cell proliferation was assessed in two human OSCC cell lines (H400 and BICR56) and in normal oral keratinocytes (OKF6) treated with the identified Kava constituents, namely Flavokawain A (FKA), Flavokawain B (FKB), yangonin, kavain and methysticin using an MTS in vitro assay. Cell migration at 16 h was assessed using a Transwell migration assay. Cell invasion was measured at 22 h using a Matrigel assay. Cell adhesion was assessed at 90 min with a Cytoselect Adhesion assay. The two Kava preparations contained substantially different concentrations of the main chemical constituents. Treatment of malignant and normal oral keratinocyte cell lines with three of the identified constituents, 10 μg/ml FKA, 2.5 μg/ml FKB and 10 μg/ml yangonin, showed a significant reduction in cell proliferation in both H400 and BICR56 cancer cell lines but not in normal OKF6 cells. Remarkably, the same Kava constituents induced a significant reduction of OSCC cell migration and invasion. We have demonstrated, for the first time, that Kava constituents, FKA, FKB and yangonin have potential anticancer effects on OSCC. This highlights an avenue for further research of Kava constituents in the development of future cancer therapies to prevent and treat OSCC.

## Introduction

Oral cancer is the sixth most common malignancy in the world^1^ with oral squamous cell carcinoma (OSCC) encompassing about 90% of oral cancers^2^. Treatment of OSCC includes single modality surgery, radiotherapy or various combinations of these modalities with or without systemic therapy (chemotherapy and/or target agents).

**Figure 1:**
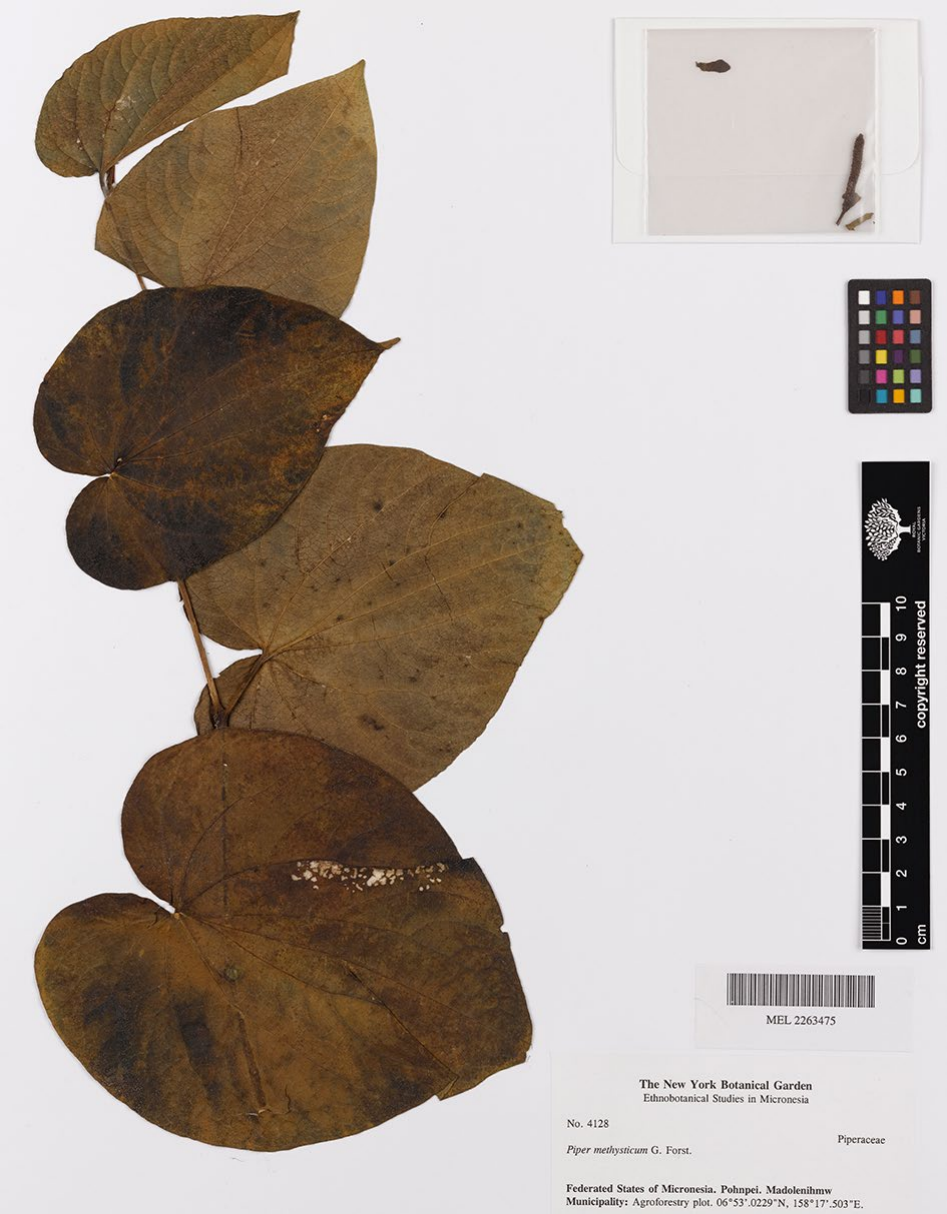
Piper Methysticum specimens. Piper Methysticum pressed plant specimens from the collection of the National herbarium of Victoria collection. Images were captured with a Leaf Aptus‐II 10 Digital Back camera. Reproduced with permission from the Royal Botanic Gardens Victoria.

Kava is a beverage made from the ground roots of the plant *Piper Methysticum* and has long held a significant place within Pacific Island communities (Fig. 1). Based on pottery and language evolution, Kava’s consumption likely dates back more than 2500 years, being used as a medicinal, social, as well as ceremonial beverage^3^. The beverage is still a popular recreational drink in Pacific Island countries akin to alcohol in western societies, inducing sedative, anaesthetic, and euphoriant effects. Conversely, Kava usage in regions of Australia and New Zealand can be attributed to the migration of Pacific Islander communities^4^. In 1982 Kava was introduced as an alcohol alternative to Australian Aboriginal communities of Arnhem Island in an attempt to reduce alcohol-related harm^5–7^. Reports of Kava hepatoxicity risk have been related to excessive consumption^8^. Studies assessing the hepatotoxicity of Kava, have identified a number of potentially contributing factors. These include the type of Kava cultivar used, impurities, and other parts of Kava used (stem and leaves) in addition to the rhizome^9^. Kava hepatotoxicity has also been associated with metabolic aberration in a few individuals^10^. Additionally, mouldy and non-mouldy contaminants in Kava extracts, and other impurities, have been suggested as potential causes of toxicity^11^. While hepatoxicity is a debateable topic and effects are not clear^12^, here we analyse the effect of single active molecules as opposed to the entire Kava extract. Active compounds extracted from Kava, and secondary metabolites, include kavalactones, chalcones, cinnamic acid derivatives and flavanones.

Cancer cells have well-established characteristics that include dysregulated proliferation, resistance to apoptosis, evasion of growth suppressors and angiogenesis activation, resulting in replicative immortality for sustained metastasis^13^. The ability of individual Kava compounds to inhibit such mechanisms has been researched. A recent systematic review published by our group^8^ identified key chalcones, such as Flavokawain B (FKB), possessing the ability to induce apoptosis, inhibit proliferation, and interfere with metastasis within multiple cancer cells, in vitro and in vivo. Flavokawain A (FKA), a separate chalcone, was also shown to induce cell cycle arrest and apoptosis in bladder and breast cancer cells^14,15^.

Our recent review also identified that research into Kava’s anti-cancer effects on OSCC is limited. The study by Hseu et al.^16^ was the only one to investigate the anti-cancer components of Kava in OSCC cells, specifically exploring the chemo-preventive effect of FKB on two human tongue OSCC cell lines.

Our current study aimed to investigate the main chemical constituents of two Kava mixtures, of varying origin (Fiji and Vanuatu). The study used gas chromatography mass spectrometry (GCMS) to characterize the commercially available Kava products. In particular, the GCMS testing identified five main Kava constituents, namely FKA, FKB, yangonin, kavain and methysticin. These constituents were subsequently tested in in vitro OSCC models to identify potential anticancer effects.

Our study showed that preparations of Kava from different origins may contain substantially different concentrations of lactones and dihydrochalcones. Our results from in vitro models have clearly demonstrated, for the first time, that three Kava constituents, FKA, FKB and yangonin exert anticancer effects on OSCC. Our findings show potential for the translation of these compounds from bench to preclinical animal models.

## Results

### Composition of Kava constituents in samples from Fiji and Vanuatu

**Figure 2:**
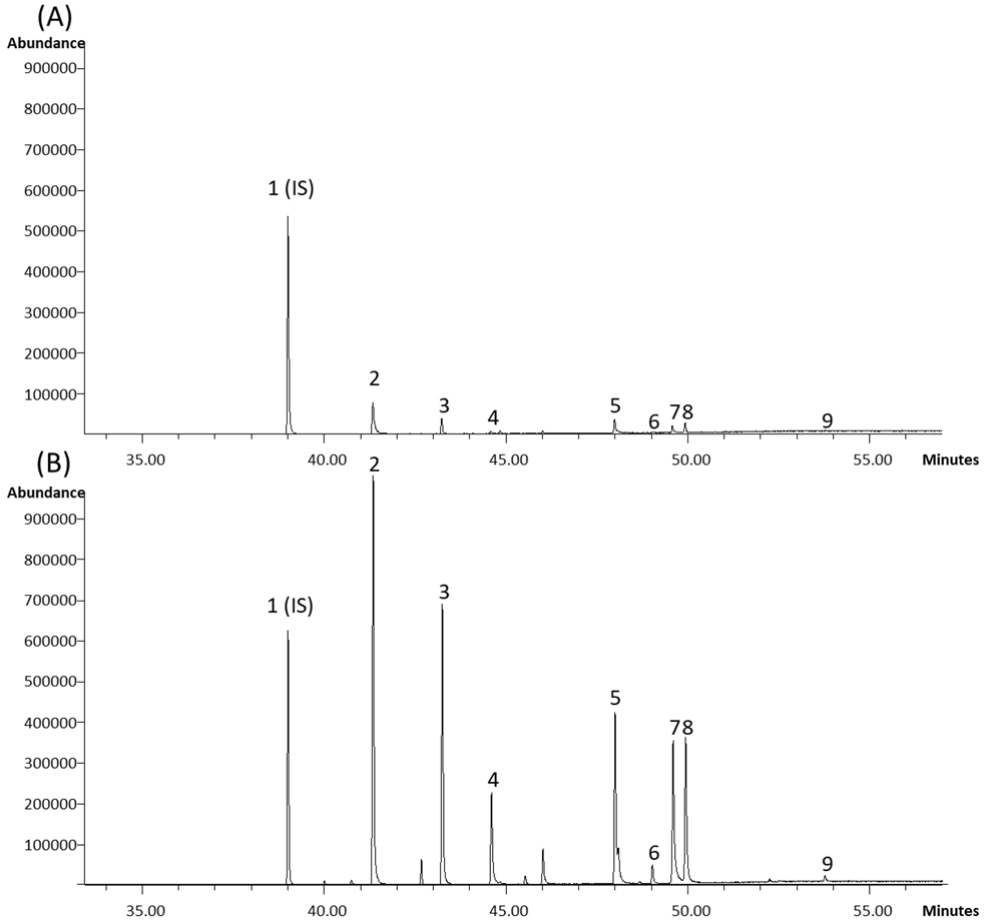
Chromatograms showing the differences in Kava constituents between two Kava samples. (**A**) Kava Fiji; (**B**) Kava Vanuatu. Peaks: (1) internal standard, methyl heptadecanoate; (2) dihydrokavain; (3) kavain; (4) desmethoxyyangonin; (5) dihydromethysticin; (6) flavokawain B; (7) yangonin; (8) methysticin; (9) flavokawain A. Flavokawain C not detected in either of the samples.

GCMS analysis was able to successfully quantify 9 chemical components belonging to the lactone and dihydrochalcone family from both mixtures. In addition to the 9 investigated constituents 3 relatively larger peaks were identified as presented in Fig. 2B. The two most researched constituents of Kava, FKA and FKB, were present in both samples, with the commercial preparation from Vanuatu showing higher concentrations compared to traditional Fijian Kava (7.68 ± 0.83 vs 0.29 ± 0.21 g/Kg and 15.14 ± 1.05 vs 0.88 ± 0.01 g/Kg, respectively) (Table 1). Interestingly, FKC was not detected in any of the mixtures (Fig. 2). The constituents investigated in this study included 5 promising active compounds namely FKA, FKB, yangonin, methysticin and kavain. Overall, preparations of Kava from different origins contained substantially different concentrations of lactones and dihydrochalcones.

**Table 1:** Composition of kavalactones and dihydrochalcones in two types of kava samples; Kava Fiji and Kava Vanuatu. Flavokawain C was not detected in either of the samples.

Peak number	Retention index	Kava constituent	Kava Fiji (g/kg)	Kava Vanuatu (g/kg)	Fiji vs Vanuatu *p* value	Target ion	Quality control Ion	Retention time (min)	Standard curve equation	R^2^
**Kavalactones**										
1	1681	Heptadecanoic acid, methyl ester				74	87, 55, 143	39.013	Internal Standard	
2	2149	(S)-(+)-7,8-Dihydrokavain	7.48 ± 0.12	29.86 ± 2.69	0.0001	127	91, 117, 232	41.349	y = − 0.0086 × 2 + 0.2416x + 0.0266	0.9998
3	2253	DL-Kavain	6.14 ± 0.22	53.57 ± 4.82	0.0001	98	68, 202, 230	43.249	y = − 0.3368 × 2 + 1.1488x + 0.0207	0.9993
4	2336	Desmethoxyyangonin	3.18 ± 0.04	13.53 ± 1.39	0.0002	228	157, 200,	44.657	y = − 0.0159 × 2 + 0.2604x + 0.0248	0.9994
5	2550	Dihydromethysticin	6.72 ± 0.11	31.11 ± 3.14	0.0002	135	276, 161	48	y = − 0.0236 × 2 + 0.3173x + 0.0284	0.9997
7	2699	Yangonin	5.92 ± 0.18	32.62 ± 0.62	< 0.0001	258	187, 230, 215	49.658	y = − 0.0343 × 2 + 0.3733x + 0.0392	0.9982
8	2716	Methysticin	6.5 ± 0.27	47.48 ± 3.9	0.0001	135	148, 274, 230	49.938	y = − 0.2405 × 2 + 1.193x + 0.0213	0.9997
Dihydrochalcones										
9	2946	Flavokawain A	0.29 ± 0.21	7.68 ± 0.83	0.0001	313	121, 207, 134	53.84	y = − 2.0474 × 2 + 3.0275x + 0.0007	0.993
6	2611	Flavokawain B	0.88 ± 0.01	15.14 ± 1.05	< 0.0001	207	283, 181, 284	49.07	y = − 0.8198 × 2 + 1.8287x—0.0028	0.9939
		Total kavalactones and Dihydrochalcones	37.1 ± 0.37	230.98 ± 18.11	< 0.0001					

### Treatment with Kava constituents; FKA, FKB and Yangonin, reduces OSCC cell proliferation

An MTS assay was performed to analyse the effect of Kava constituents; FKA, FKB, yangonin, methysticin and kavain on OSCC cells (H400, BICR56), and human oral epithelial cell line OKF6.

**Figure 3:**
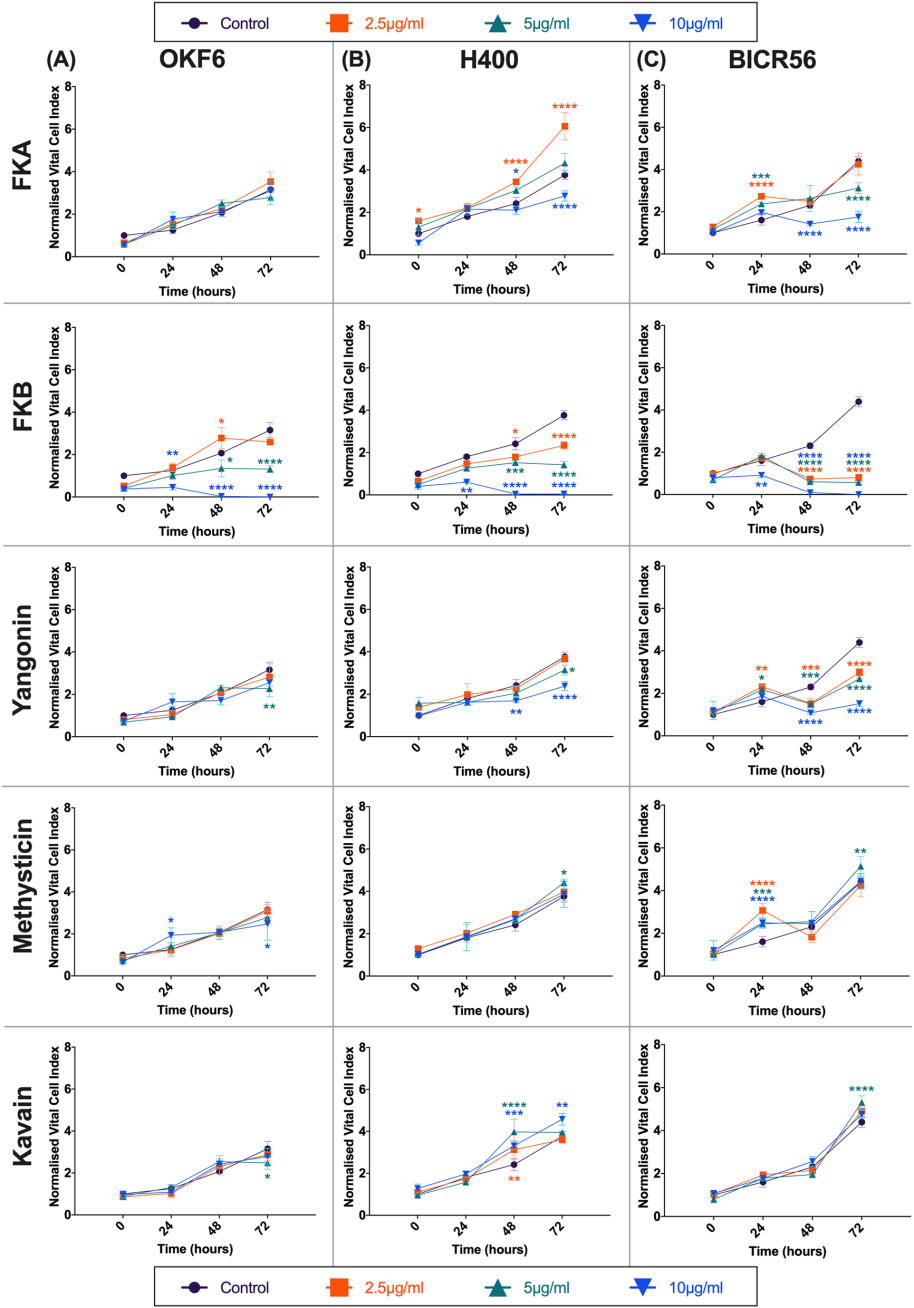
MTS assay. the effect of 2.5 μg/ml, 5 μg/ml and 10 μg/ml of FKA, FKB, yangonin, methysticin and kavain on OKF6 (**A**), H400 (**B**) and BICR56 (**C**) cell proliferation. Data are represented as mean ± SD. Statistical significance is given as follows: *p < 0.05, **p < 0.01, ***p < 0.005, ****p < 0.001; compared to control group.

Treatment with FKA, in the H400 cell line, significantly reduced cell growth at a concentration of 10 μg/ml, at timepoint 72 h. Similar results were also noted in BICR56 cells as early as 48 h. FKA did not exert any significant reduction of cell growth in OKF6 cells. Low concentrations of FKA promoted OSCC cell growth. This was evident as early as 48 h in the H400 cell line and at 24 h in BICR56 cell line (Fig. 3).

OSCC cells treated with a low concentration of FKB, 2.5 μg/ml, confirmed a significant reduction of cell growth in both the H400 and BICR56 cell lines. 2.5 μg/ml of FKB did not present any significant reduction of cell growth in OKF6 cells but did indicate a significant increase at 48 h (Fig. 3). High concentrations of FKB indicated cytotoxicity in all cell lines as early as 48 h at a concentration of 5 μg/ml and as early as 24 h at a concentration of 10 μg/ml (Fig. 3).

Yangonin, at a high concentration of 10 μg/ml significantly reduced cell growth both H400 and BICR56 cell lines as early as 48 h. Cell growth was unaffected in OKF6 cells. The BICR56 cell line treated with 2.5 μg/ml and 5 μg/ml of yangonin, experienced a significant reduction of growth as early as 24 h (Fig. 3). Whereas the H400 cell line experienced a significant reduction of growth only at 72 h when treated with 5 μg/ml. In OKF6 cell line, treatment with yangonin at a concentration of 5 μg/ml induced a significant reduction in cell growth at 72 h (Fig. 3).

Treatment with kavain, in H400 cell line, significantly increased cell growth at concentrations of 2.5 μg/ml at 48 h, 5 μg/ml at 48 h and 10 μg/ml as early as 48 h (Fig. 3). Significant cell growth was only indicated in the BICR56 cell line at a concentration of 5 μg/ml at 72 h. Similarly, 5 μg/ml of kavain in the OKF6 cell line significantly increased cell growth after 72 h (Fig. 3).

Methysticin treated BICR56 cells significantly increased growth at concentrations of 2.5 μg/ml and 10 μg/ml at 24 h. BICR56 cell line treated with 5 μg/ml, significantly increased growth at 24 and 72 h (Fig. 3). Similarly, H400 cell line treated with 5 μg/ml significantly increased growth at 72 h. OKF6 cell line treated with 10 μg/ml of methysticin, significantly increased growth at 24 and 72 h (Fig. 3). Overall, our data show that the anti-proliferative effects of Kava constituents were concentration-specific and had greater effect in OSCC cells compared to normal OKF6 cells.

### Treatment with Kava constituents; FKA, FKB and Yangonin, induces a reduction in OSCC cell migration and invasion

**Figure 4:**
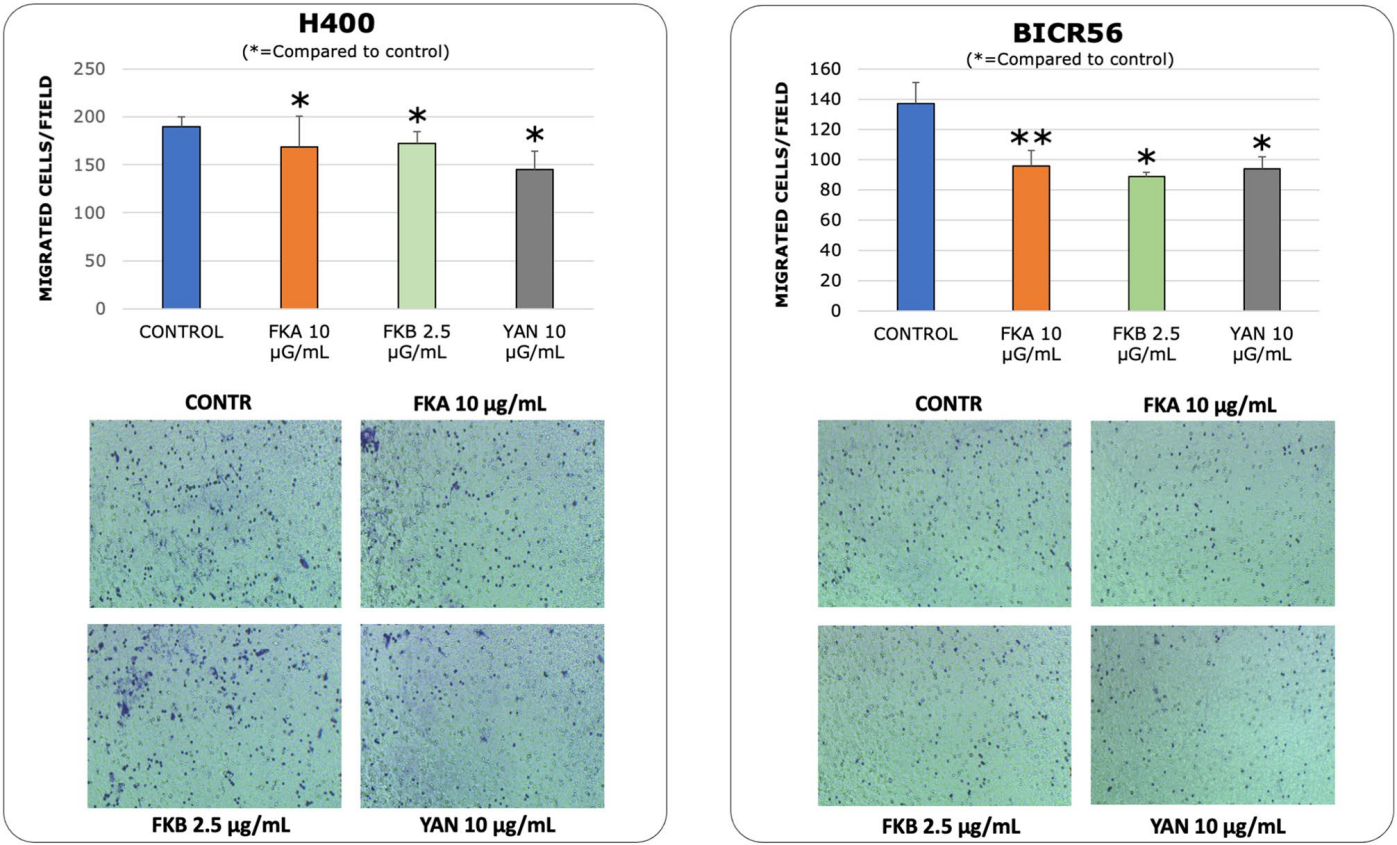
Migration assays. migrated cell count of H400 and BICR56 cells incubated with 10 μg/ml FKA, 2.5 μg/ml FKB and 10 μg/mL yangonin. The results were representative of 3 independent experiments. The representative microscopic fields showed (digital white light microscopy, 460 × magnification) are H400 and BICR56 migrated cells at 16 h when treated with no treatment (control), 10 μg/ml FKA, 2.5 μg/ml, FKB and 10 μg/mL yangonin. Data are represented as mean ± SD. Statistical significance is given as follows: *p < 0.05; **p < 0.005; compared to control group.

**Figure 5:**
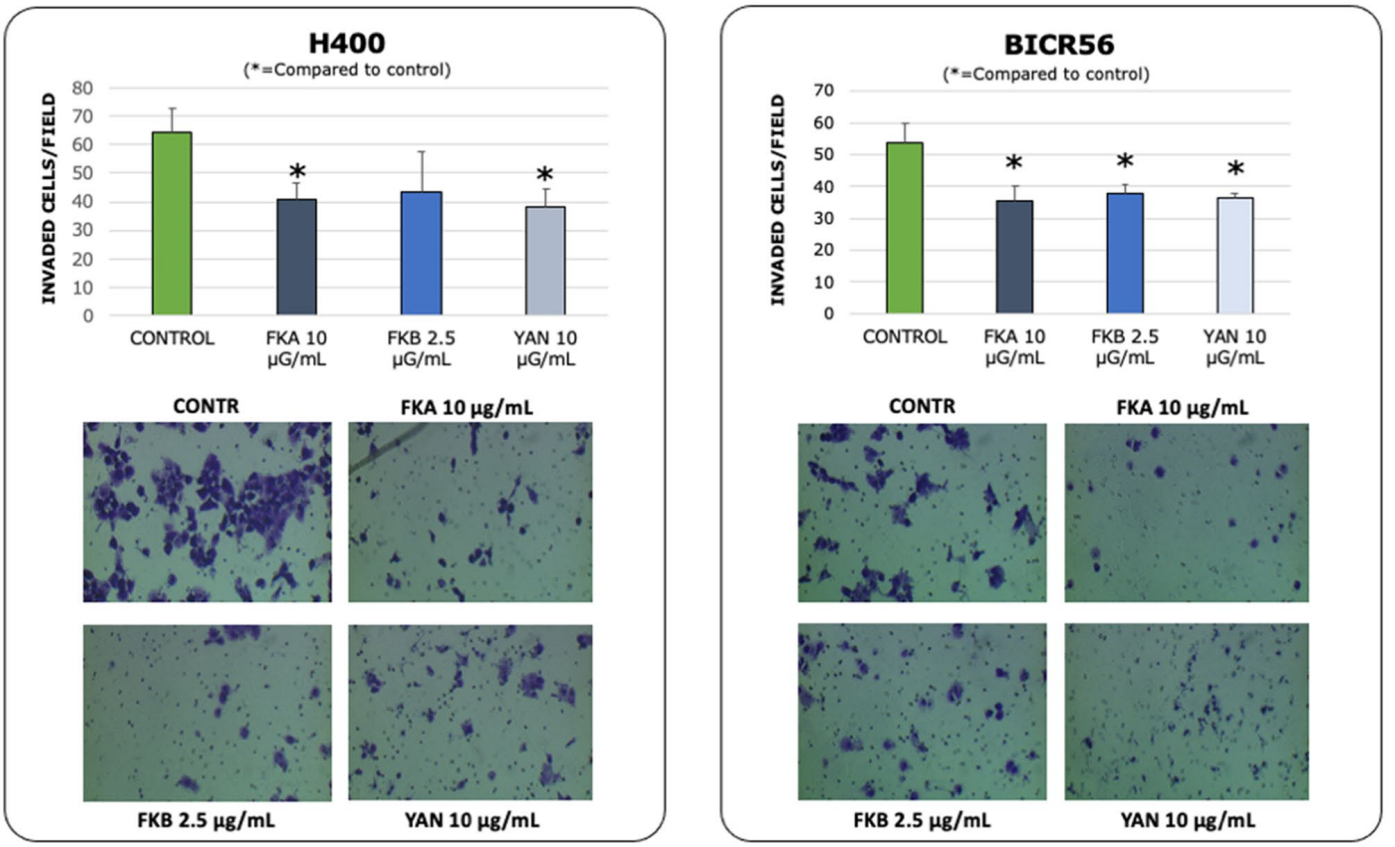
Invasion assays. invaded cell count of H400 and BICR56 cells incubated with 10 μg/ml FKA, 2.5 μg/ml FKB and 10 μg/mL yangonin. Images were taken after 22 h at × 20 magnification (optical white light microscopy) showing fewer invading cells for treated groups compared to the control (representative fields). Graphs show quantification of invasion assays. The results were representative of 3 independent experiments. Data are represented as mean ± SD. Statistical significance is given as follows: *p < 0.05; compared to control group.

The concentrations of Kava constituents which significantly reduced H400 and BICR56 cell growth were selected to assess cell migration and invasion in vitro. These included FKA, FKB and yangonin at concentrations of 10 μg/ml, 2.5 μg/ml and 10 μg/ml, respectively. Serum-free medium in the upper chamber and a tailored timepoint (< 24 h) was used to minimise the effect of cell proliferation. Both the H400 and BICR56 cell lines showed a significant reduction in cell migration at 16 h when treated with either FKA, FKB or yangonin, compared to the control (Fig. 4). Similarly, both the H400 and BICR56 cell lines showed a significant reduction in cell invasion at 22 h when treated with either FKA or yangonin, compared to the control. However, FKB demonstrated a significant reduction in the ability to invade for only the BICR56 cell line (Fig. 5).

### Treatment with Kava constituents; FKA, FKB and Yangonin, does not alter OSCC adhesion

**Figure 6:**
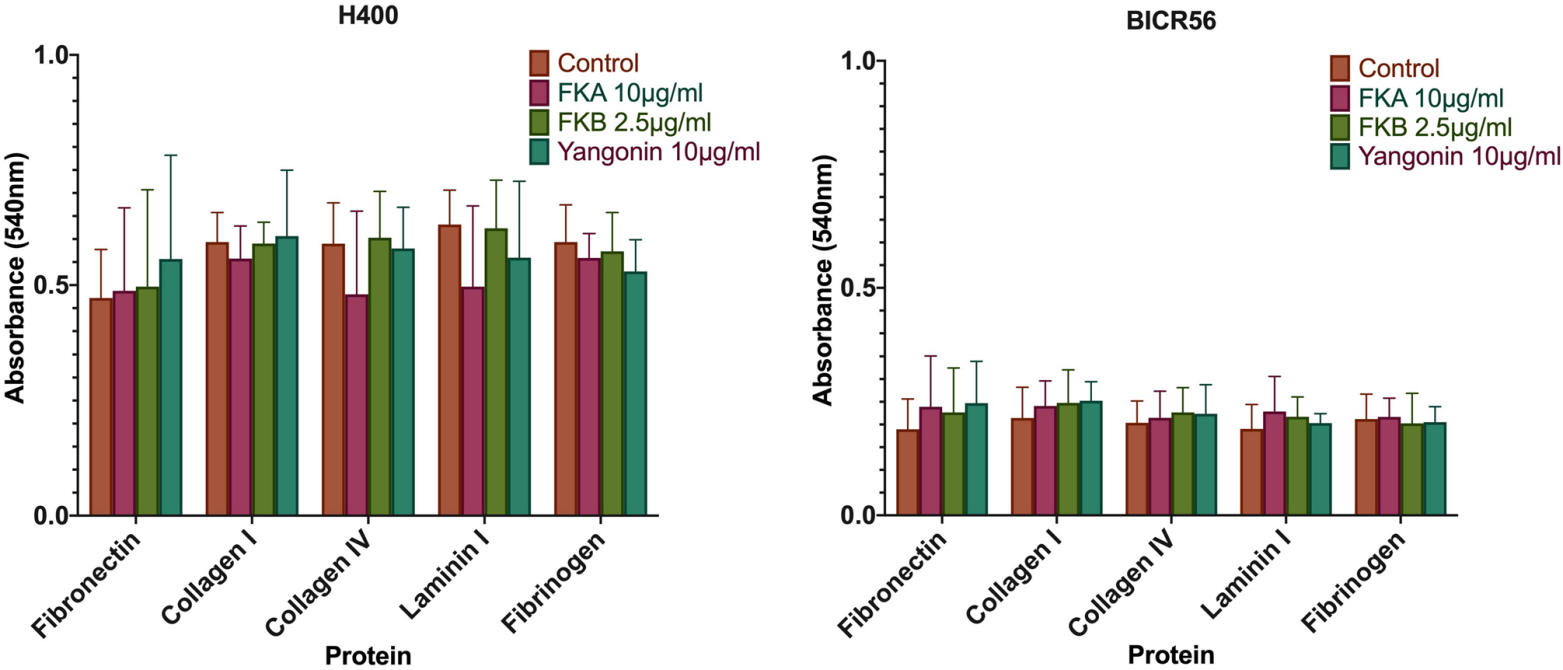
Adhesion assay. the effect of 10 μg/ml FKA, 2.5 μg/ml FKB and 10 μg/mL yangonin on H400 and BICR56 cells. Data are represented as mean ± SD. Statistical significance is given as follows: *p < 0.05, **p < 0.01, ***p < 0.005, ****p < 0.001; compared to control group.

Selective focal adhesion of H400 and BICR56 to ECM molecules was not significantly affected when incubated with Kava constituents; FKA, FKB and yangonin, in comparison to the control. ECM molecules tested were fibronectin, collagen I, collagen IV, laminin I or fibrinogen (Fig. 6).

## Discussion

In the present study, we demonstrate that treatment of OSCC cells with Kava constituents; FKA, FKB and yangonin, affects cell proliferation, migration and invasion. Literature has presented constituents of Kava as having chemotherapeutic potential in cancer prevention and treatment. Our results are the first to demonstrate the possible use of Kava constituents in the treatment of OSCC.

Literature indicates FKA to induce apoptosis^14,15,17–19^ and inhibit proliferation^14,17–20^. Additional anticancer findings involve the prevention of metastasis, inhibition of angiogenesis, reduction in inflammation and enhancement of immune function^14,17,19^. In the present study, we show that treatment of FKA affects OSCC cell proliferation. Cells treated with low concentrations of FKA exhibited a promoting effect of cell proliferation in both cancer cell lines. However, at 10 μg/ml FKA exerted an anti-proliferative effect, where the same concentration did not affect human normal oral epithelial OKF6 cells. This finding has salient clinical implications as it suggests that the effects of FKA are specific to oral malignant cells. Further investigation into 10 μg/ml FKA demonstrated a reduction in ability of OSCC migration and invasion and thus a potential to reduce malignant hallmarks of cancer. Our data show that 10 μg/ml of FKA is a targetable concentration for treatment of oral carcinogenesis.

The literature shows FKB to have an antiproliferative ability^13,15,16,21–27^ and to induce apoptosis^13,16,20–25,27–32^. Similar to FKA, studies have indicated the prevention of metastasis^13,28,33^, anti‐angiogenic effects^13,33^, as well as regulation of immune and inflammatory functions^28^. Our study has demonstrated high concentrations of FKB to be cytotoxic to all cell lines. However, low concentrations of 2.5 μg/ml of FKB were cytotoxic to OSCC cell lines H400 and BICR56 but had no effect on human oral epithelial cell line OKF6. Additional investigation into this concentration of FKB demonstrated a reduction in ability of OSCC migration and cancer cell dependent reduction in invasion. This indicates that 2.5 μg/ml of FKB may be a promising anti-cancer target as it did not affect normal OKF6 cells.

Yangonin, a minimally researched component of Kava, has demonstrated variable findings in the literature to inhibit growth^34,35^. Various studies of conflicting results motivated us to investigate the anti-cancer properties of yangonin. The results of our study are the first to identify the cytotoxicity of high concentrations of yangonin (10 μg/ml) in OSCC cells. Low concentrations are cancer cell dependent. Similar to FKA, assessment of 10 μg/ml of yangonin demonstrated a reduction in ability of OSCC migration and invasion and thus a potential to reduce malignant hallmarks of cancer. Our data demonstrates that yangonin is a targetable Kava constituent for treatment of oral carcinogenesis, however, further tests are required on additional cancer cell types.

The limited research on kavain’s anti-cancer properties showed conflicting results. Various studies have shown its ability to reduce cellular growth of cancer cells^34,36^, while others have demonstrated minimal or lack of ability to effect proliferation or apoptosis^20^. These contrasting findings were found on bladder cancer cells, where the epithelial lining of the bladder shares similarities to the lining of the oral cavity. Our in vitro model of OSCC, treated with kavain, stimulates cancer cell proliferation in both cell lines at all concentrations. It did not however affect cell proliferation in human oral epithelial cell line OKF6. Kavain is therefore a potential cancer promoting agent.

Methysticin, similar to yangonin, has limited research examining its anticancer properties. Although studies suggest methysticin may have anticancer properties, the concentrations tested and reported potency were variable between studies^33,34,37^. The variability between studies indicates the need for further research. Our in vitro model of OSCC, treated with methysticin, indicated a stimulation of cancer cell proliferation which was potentially cancer cell specific. Cell proliferation was also observed in human oral epithelial cell line OKF6. Kava constituent methysticin should be tested on additional cancer cell types.

In conclusion, we have shown that preparations of Kava from different origins may contain substantially different concentrations of lactones and dihydrochalcones. Kava constituents; FKA, FKB and yangonin reduce cell proliferation in a time, dose-specific manner in both OSCC cell lines tested but not in normal oral keratinocytes. Additionally, reduction of cell migration of FKA, FKB and yangonin appears to be dose specific but irrespective of OSCC cell line tested. This was also evident in the reduction of cell invasion of FKA and yangonin. The reduction of cell invasion of yangonin appears to be dose and cell line specific. FKA, FKB and yangonin appear to have no effect on cell adhesion to ECM proteins. We have demonstrated, for the first time, that Kava constituents; FKA, FKB and yangonin have potential antiproliferative effects on OSCC with a difference in normal and malignant tissues. These Kava constituents show promising results for the treatment of oral carcinogenesis and a translation into in vivo studies. Further research into the OSCC protein binding and the mechanisms of action are encouraged.

## Methods

All experiments were performed in accordance with relevant guidelines and regulations as approved by the Research Ethics Committee of the University of Melbourne (Project 1340716.1).

### Gas chromatography mass spectrometry (GCMS)

Kava samples were prepared for lactone and dihydrochalcone analysis based on published protocols with modifications^38,39^. For each Kava sample, 100 mg of powdered Kava sample was sub-sampled, and extracted three times with 3 ml of acetone by sonication for 30 min. The extracts were combined and adjusted to 10 ml in volumetric flasks and filtered through a 0.45 µm membrane. 0.9 ml of sample was transferred to a 2 ml Gas Chromatography (GC) vial and mixed with 20 µl of methyl heptadecanoate as an internal standard (IS) (1.7 mg/ml in acetone). All samples were prepared in triplicates.

An Agilent Technologies 6850 series II GC system (Agilent Technologies, Santa Clara, CA) connected to an Agilent Technologies 6973 mass selective detector (MSD) and coupled with an Agilent PAL multipurpose autosampler were used for volatile analysis. The instruments were controlled using Agilent G1701EA MSC ChemStation software in conjunction with Agilent PAL autosampler Control software B.01.04 for ChemStation. The GC was fitted with a J&W DB-5 ms column (30 m × 0.25 mm, 0.25 µm film df, with helium as carrier gas (Ultrahigh Purity, BOC Australia, North Ryde, NSW, Australia). The GC inlet was fitted with a borosilicate glass split inlet liner (volume 935 µl, Agilent Technologies) and held at 250 °C. 1 µl of sample was injected into the inlet in split mode at a split ratio of 30:1, and a constant column flow rate of 1 ml/min. The column was held at 50 °C for 6 min before increasing to 280 °C at 5 °C/min, held at 280 °C for 5 min, and post run at 50 °C for 1 min. The MS source, quadruple and transfer line were held at 230 °C, 150 °C, and 280 °C, respectively. The MS was operated in scan mode (35–350 m/z) with positive EI of 70 eV. The standard solution of dihydrokavain, kavain, desmethoxyyangonin, dihydromethysticin, yangonin and methysticin were prepared at 6 scale dilutions from 20 to 1000 mg/L in acetone, while flavokawain A, flavokawain B and flavokawain C were prepared at 7 scale dilutions from 10 to 1000 mg/L in chloroform. All standard solutions were spiked with internal standard and analyzed as sample to generate standard curves (R^2^ > 0.99). Kavalactones and dihydrochalcones were identified by comparing the mass spectra and retention indices with the NIST library in ChemStation and Webbook database and the standard solutions obtained. All compounds were quantified based on the standard curves using target ions. Blank and internal standards were checked regularly to ensure the sensitivity of GCMSD system.

### Cell lines

Two human malignant oral keratinocyte cell lines derived from different intra-oral sites, H400 and BICR56, and one normal human oral keratinocyte cell line, OKF6 were selected for this study. The OSCC adherent cell lines were established at Bristol Dental School, University of Bristol, UK^40^, from primary explants of tongue (BICR56) and alveolar process (H400) squamous cell carcinoma. All OSCCs were HPV-negative and were authenticated prior to commencing the experiments.

### Culture conditions

The OSCC cell lines were cultured as previously described^41^. Cell lines were cultured in 100-mm Petri plastic dishes (Corning 430167) and grown to 60%-80% confluence before being further sub-cultured. Cells were cultured using Dulbecco's modified Eagle's medium (DMEM) (D5796) and nutrient mixture F-12 Ham (N6658) in a 1:1 ratio (Sigma-Aldrich, Australia), supplemented with 10% foetal bovine serum (FBS) (SFBS-F, Bovogen, Keilor East, Vic, Australia), 1% penicillin streptomycin mixture (P4333, Sigma-Aldrich, Castle Hill, NSW, Australia) and 0.5 μg/mL hydrocortisone (HC) (H6909, Sigma-Aldrich, Castle Hill, NSW, Australia) in a humidified atmosphere at standard conditions (5% CO2, 37 °C). The normal human oral mucosal epithelial cell line OKF6, was instead cultured in 100-mm Petri plastic dishes (Corning 430167, Corning, NY, USA) and grown to 60–80% confluence before being further sub-cultured. OKF6 cells were cultured using keratinocyte serum-free medium (K-SFM) (#17005‐042, Thermo Fisher Scientific) containing 0.4 μg/ml bovine pituitary extract and 0.2 ng/ml human recombinant epidermal growth factor (as per manufacturer’s instructions), 0.3 mM CaCl_2_, 1% penicillin streptomycin mixture (P4333, Sigma-Aldrich, Castle Hill, NSW, Australia) and supplemented with 1% Newborn Calf Serum (NCS) (N4637, Sigma-Aldrich, Castle Hill, NSW, Australia). OKF6 cells were incubated at 37 °C, 5% CO_2_ for 5 to 7 days to reach 80% confluency.

Epithelial cells grown to 80% confluency were subsequently detached via a pre-treatment of 10 mM EDTA for 10 min, followed subsequently with incubation with a 0.25% trypsin in 1 mM EDTA solution (T4049, Sigma-Aldrich, Castle Hill, NSW, Australia) for 5 min. The viability of the keratinocytes was confirmed by trypan blue exclusion (trypan blue dye, 0.4% solution, 1450021, Bio-Rad)^41^.

### Kava constituents

FKA, FKB, yangonin, methysticin and kavain (LKT Laboratories, USA) were all reconstituted in Dimethyl Sulfoxide (DMSO) to respective concentrations of 31.44 mg/mL, 10 mg/mL, 10 mg/mL, 10 mg/mL, and 12.5 mg/mL.

### Proliferation assays

Analysis of H400, BICR56 and OKF6 cells was performed by seeding cells in 96-well plates overnight and treating with 2.5 μg/ml, 5 μg/ml and 10 μg/ml of FKA, FKB, yangonin, methysticin or kavain. Cell viability was measured using MTS cell proliferation assay kit (CellTiter 96 AQueous One Solution, G3580, Promega, Madison, USA) as per manufacturer's instructions. Absorbance was measured at 490–500 nm at timepoints 0, 24, 48 and 72 h (Wallac Victor 3, Perkin Elmer). All the experiments were performed in triplicate.

### Migration assay

In vitro cell migration of H400 and BICR56 cells was assessed at 16 h using a Transwell migration assay (CLS3422, Sigma-Aldrich) as previously described^41^. Kava constituents FKA (10 μg/ml), FKB (2.5 μg/ml) and yangonin (10 μg/ml) were used to treat the cells in the upper chambers of the assay. 1.5 × 10^4^ cells/well were seeded in serum-free media. A culture medium with 10% FBS without antibiotics was used in the lower chamber. After incubation in standard conditions (37 °C, 5% CO_2_), the medium was removed. The cells in the upper chamber were fixed in 4% formalin for 2 min at room temperature (r/t), permeabilised with 100% ice-cold methanol for 20 min at room temperature and stained (UV protected) using 2% crystal violet solution for 20 min. After the removal of the non-migrated cells, the migrated cells (5 random fields/well) were observed under a BH2 Olympus microscope equipped with a PMW-10MD Sony camera. Captured images were analysed to count migrated cells using ImageJ Software (ImageJ v. 1.50i, National Institutes of Health). All the experiments were performed in triplicate.

### Invasion assay

In vitro cell invasion of H400 and BICR56 cells was assessed at 22 h using a Corning BioCoat Matrigel Invasion Chamber assay (Corning 354480) as previously described^41^. Kava constituents FKA (10 μg/ml), FKB (2.5 μg/ml) and yangonin (10 μg/ml) were used to treat the cells in the upper chambers of the assay. Cells were seeded with a density of 3.0 × 10^5^ cells/well. A culture medium with 10% FBS without antibiotics was used in the lower chamber as chemoattractant. After incubation in standard conditions for 22 h, the medium was removed. The cells in the upper chamber were fixed with 100% ice-cold methanol for 2 min at room temperature and stained (UV protected) using 2% crystal violet solution for 2 min, as per manufacturer's instructions. After the removal of the non-migrated cells, the migrated cells (5 random fields/well) were observed under a BH2 Olympus microscope equipped with a PMW-10MD Sony camera. Captured images were analysed to count migrated cells using ImageJ Software (ImageJ v. 1.50i, National Institutes of Health). All the experiments were performed in triplicate.

### Adhesion assay

In vitro cell adhesion assay of H400 and BICR56 cells was assessed at 90 min using a CytoSelect 48-Well Cell Adhesion Assay (Cell Biolabs, Inc., CBA-070) according to the manufacturer’s instruction. Kava constituents FKA (10 μg/ml), FKB (2.5 μg/ml) and yangonin (10 μg/ml) were used to treat the cells in a serum-free culture medium without antibiotics. 5.0 × 10^4^ cells/well were seeded. Absorbance was measured at 540 nm (Wallac Victor 3, Perkin Elmer). All the experiments were performed in triplicate.

### Statistical analysis

Quantification of kavalactones and dihydrochalcones by GCMS was compared using ANOVA and post-hoc Tukey’s test with significance determined by *p* < 0.05 in CoStat Software (version 6.4, CoHort software, Monterey, USA). Differences between groups in the cell proliferation/adhesion assays were analysed using two-way ANOVA test comparing time point, concentration of drug and absorbance using GraphPad Prism 8.0.1 (La Jolla, California, USA). Cell migration and invasion assay data was analysed by variables using t test, ANOVA, and Fisher's exact test and linear models with significance defined as *p* < 0.05 using IBM SPSS statistical software version 21.0 and R 3.3.3.
